# Social support and self-management activation among older adult chronic disease patients in China: the chain mediating role of acceptance of illness and fatigue

**DOI:** 10.3389/fpubh.2025.1637017

**Published:** 2025-08-12

**Authors:** Shi Qing Zhang, Man Deng, Xue Jun Xu, Yue Yang, Shuang Zhao, Jianxin Yue, Yong Xia Chen, Fu Zhi Wang, Xiumu Yang

**Affiliations:** ^1^School of Nursing, Bengbu Medical University, Bengbu, China; ^2^School of Nursing, Anhui Medical University, Hefei, China; ^3^Department of Nursing, Bengbu First People's Hospital, Bengbu, China; ^4^Department of Rehabilitation, The Second Affiliated Hospital, Bengbu Medical University, Bengbu, China; ^5^Department of Nursing, First People's Hospital Affiliated with Bengbu Medical University, Bengbu, China; ^6^School of Health Management, Bengbu Medical University, Bengbu, China; ^7^General Practice Education and Development Center, Bengbu Medical University, Bengbu, China

**Keywords:** social support, acceptance of illness, fatigue, self-management activation, mediation

## Abstract

**Background:**

With the rapid aging of the global population and the continuous increase in the incidence of chronic diseases among the older adult, self-management activation has become a key factor in improving patients’ quality of life. This study examines the relationship between social support and self-management activation in older adult patients with chronic diseases, with a focus on the mediating roles of illness acceptance and fatigue within this framework.

**Methods:**

A convenience sampling method was employed to recruit 317 older adult patients with chronic diseases from three communities in Hefei, Anhui Province, China, between August and November 2023. Data collection involved the use of a general information questionnaire, the Illness Acceptance Scale, the Self-Rating Social Support Scale, the Chinese version of the Multidimensional Fatigue Inventory, and the Public Health Activation Index Scale. The influencing factors were examined through independent t-tests, one-way ANOVA, and Pearson correlation analysis in SPSS 26.0. Additionally, a structural equation model (SEM) in AMOS 26.0 was employed to evaluate the possible mediating roles of acceptance of illness and fatigue in the connection between social support and self-management activation.

**Results:**

A strong positive relationship was observed between social support, acceptance of illness, and Self-management activation (*r* = 0.615 and 0.787, *p* < 0.01). In contrast, a significant negative correlation was observed between fatigue and self-management activation (*r* = −0.695, *p* < 0.01). Further analysis using structural equation modeling (SEM) demonstrated that the model exhibited a good fit (RMSEA = 0.076, *p* < 0.05). The results indicated that acceptance of illness and fatigue significantly mediate the connection between social support and self-management activation. Mediation effect analysis revealed that the direct impact of social support, acceptance of illness, and fatigue on self-management activation accounted for 24.2% of the overall effect. In comparison, the indirect effects made up 75.8%.

**Conclusion:**

This study demonstrates that the Self-management activation of older adult adults with chronic conditions is generally low and may be influenced by various individual and environmental factors. The findings highlight that enhancing acceptance of illness and alleviating fatigue symptoms are crucial intervention strategies to improve Self-management activation, particularly for patients with low levels of social support.

## 1 Introduction

According to data from the World Health Organisation (WHO), around 41 million people die from non-communicable diseases (NCDs) annually, representing 74% of global deaths (1). The older adult population is classified as a high-risk group for chronic non-communicable diseases (chronic diseases, NCDs). As China’s aging population accelerates, it is estimated that approximately 75% of individuals aged 60 and older are affected by one or more NCDs (2). NCDs are characterized by prolonged disease progression, high incidence rates, challenges in control, and substantial economic burdens, thereby posing a significant threat to public health security in China. These diseases not only pose a serious threat to individual health but also impede social and economic development, thereby presenting greater challenges to the self-management of chronic diseases. Self-management activation is defined as an individual’s awareness, knowledge, skills, and confidence in managing one’s health and healthcare. It serves as a key indicator of an individual’s proactive involvement in health management (3). Studies have demonstrated that improving self-management activation is associated with better health and disease management outcomes, increased efficiency in healthcare resource utilization, reduce misuse of healthcare resources, and ultimately lower treatment costs (4, 5).

Social support is regarded as an essential external resource for enhancing self-management activation (6). It refers to emotional comfort, material assistance, and informational support perceived by individuals as coming from family members, friends, and community organizations, which can alleviate psychological stress and enhance patients’ confidence and motivation in coping with their illness (7). Older adult patients with chronic diseases are susceptible to negative emotions, including anxiety, depression, and loneliness, due to physical decline, comorbidities, and reduced social participation, resulting in a lower willingness to engage in self-management. According to the social support buffering theory, social support can effectively mitigate the adverse effects of stress events, enhance communication and interaction with the external environment, and provide emotional regulation capabilities (8), thereby promoting self-management motivation. Therefore, the hypothesis is proposed that social support is significantly associated with self-management motivation (Hypothesis 1: Social support → Self-management activation).

Additionally, acceptance of illness refers to the extent to which patients acknowledge the facts of their illness and demonstrate psychological adaptability, playing a crucial role in chronic disease management (9). Empirical evidence indicates that acceptance of illness is a core component of disease adaptation and health behavior decision-making processes, serving as a significant predictor of self-management capacity (10, 11). Furthermore, research suggests that social support enhances illness acceptance and influences disease cognition and emotional regulation through this acceptance, thus improving patients’ health behavior intentions (12). Consequently, it is hypothesized that acceptance of illness functions as a mediating variable between social support and self-management activation in older adult patients with chronic illnesses (Hypothesis 2: Social Support → Acceptance of illness → Self-management activation).

Meanwhile, fatigue is a significant factor that hinders self-management capacity in older adult patients with chronic diseases (13). Fatigue is a complex, subjective feeling of physical exhaustion, marked by persistent reductions in energy, impaired concentration, and decreased behavioral motivation, which are difficult to relieve with rest (14). Empirical evidence suggests a negative correlation between fatigue levels and self-management engagement (15). Post-stroke fatigue can decrease medication adherence and participation in rehabilitation exercises, thus reducing involvement in disease management and social functioning, ultimately weakening self-management motivation (16). Social support has been shown to effectively reduce fatigue in patients with chronic diseases (17). Therefore, it is hypothesized that fatigue mediates the relationship between social support and self-management activation in older adult patients with chronic illnesses. (Hypothesis 3: Social support → Fatigue → Self-management activation).

Notably, previous studies have identified a significant negative correlation between the acceptance of illness and fatigue (18). Patients with higher sickness acceptance can enhance their psychosocial adaptability, reduce emotional exhaustion, alleviate anxiety and depression, and other factors contributing to mental fatigue, thus indirectly improving the sustainability of self-management intentions (19–21). Therefore, it is hypothesized that social support exerts its effects through a series of mediators, namely disease acceptance and fatigue symptoms. (Hypothesis 4: Social support → Acceptance of illness → Fatigue → Self-management activation).

Through literature review and theoretical analysis, it was found that social support, acceptance of illness, and fatigue are significantly correlated with self-management activation. However, research on the relationship pathways between them remains limited. Identifying the potential mechanisms through which social support influences self-management positivity is crucial for accurately assessing patients’ health management potential (22). Therefore, the theoretical framework of this study is based on Social Cognitive Theory (SCT), which emphasizes that when individuals face adversity or challenges, their cognition, emotions, and behavior interact with their environment, and environmental factors also play a role in individuals’ behavior and cognition, i.e., the environment, the individual, and behavioral responses interact with each other (23).

**Figure 1:**
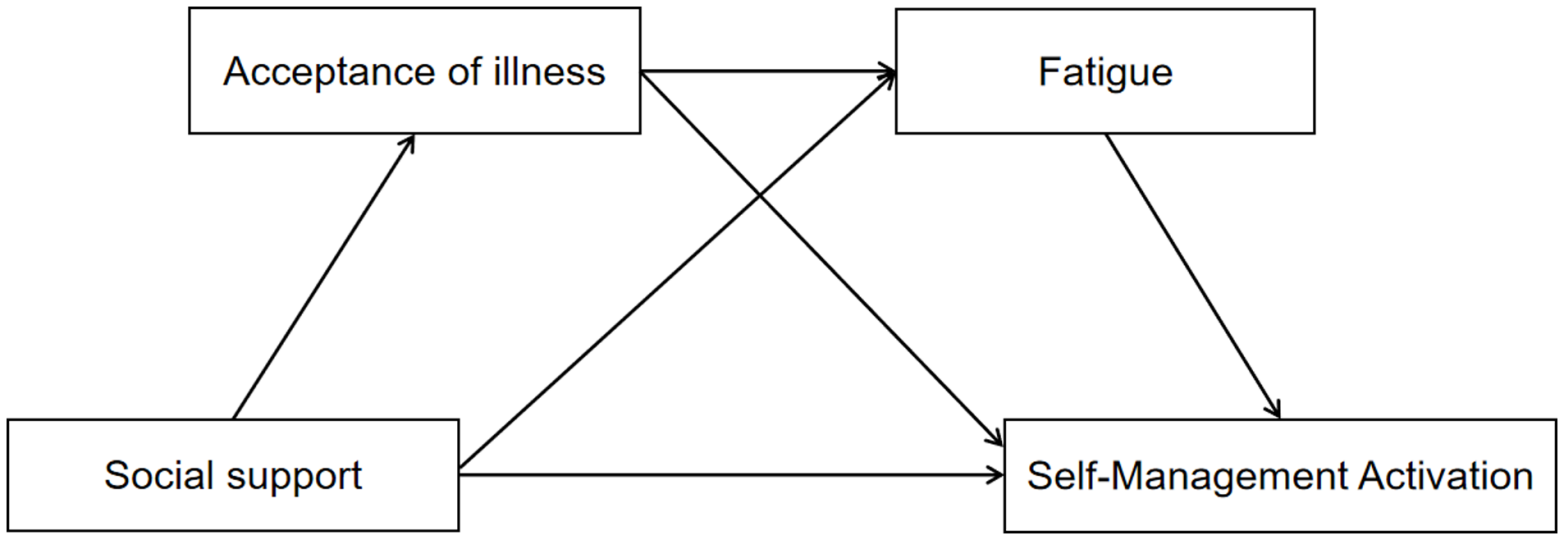
Hypothesized relationships between social support, acceptance of illness, fatigue symptoms, and self-management activation.

Specifically, when a disease impacts an individual, patients engage in cognitive appraisal of the illness and exhibit psychological stress responses. Self-management activation serves as an active stress-coping behavior, with social support regarded as a critical external protective factor. It not only offers emotional and practical assistance but also alleviates adverse effects and enhances disease-coping capacity, thereby directly promoting improvements in self-management activation (24). Acceptance of illness functions as a vital personal psychological protective factor within the Social Cognitive Theory (SCT). When confronting health challenges, acceptance of illness enables older adult patients with chronic conditions to adopt a rational and tolerant attitude toward their illness, accept its adverse effects, and strengthen psychological resilience, perceived control, and coping skills, which in turn sustain intrinsic motivation and the continuity of proactive self-management behaviors (25). Fatigue, a crucial physiological factor in SCT, manifests as reduced energy levels and impaired decision-making capacity during fatigue states, which subsequently affects self-management activation (16). Therefore, within the SCT framework, social support influences self-management activation among older adult patients with chronic diseases through the combined effects of psychological factors (acceptance of illness) and physiological factors (fatigue). This study aims to elucidate the mechanisms by which social support affects self-management activation, employing acceptance of sickness and fatigue as mediators. It constructs a multiple mediation model to explore the mediating roles of acceptance of illness and fatigue in the relationship between social support and self-management activation, thereby addressing existing research gaps and providing a theoretical basis for interventions aimed at enhancing self-management among older adult populations with chronic diseases. The conceptual framework (Figure 1) and research hypotheses are developed based on SCT.

## 2 Methods

### 2.1 Study subjects

This study is a cross-sectional design. The target population for this study was older adult individuals with chronic diseases residing in urban communities in China. A convenience sampling method was used to recruit 317 participants from three community health service centers in Hefei City, Anhui Province, between August and November 2023. Inclusion criteria: (1) Age ≥ 65 years; (2) Diagnosis of at least one chronic disease by a medical institution at the second level or higher, according to the ICD-10 criteria, such as hypertension, hyperlipidemia, diabetes mellitus, chronic obstructive pulmonary disease, asthma, chronic bronchitis, coronary heart disease, gout/hyperuricemia, cataracts, cerebrovascular disease, cirrhosis, chronic gastrointestinal disease, chronic osteoarthritic disease, and chronic kidney disease, from the 14 most common chronic diseases;(3) Community residents with a residence duration of ≥6 months; (4) Clear consciousness and ability to communicate or read usually; (5) Informed consent and voluntary participation. Exclusion criteria: (1) Requiring long-term bed rest due to trauma or hemiplegia; (2) In the acute phase of a disease or with severe organic lesions of the heart, lungs, kidneys, etc.;(3) Individuals with severe cognitive, language, hearing, or mental disorders; (4) Individuals currently participating in other studies. The sample size was estimated using G*Power 3.1 software,and the linear model calculation method (26). with a moderate effect size (f^2^ = 0.15), an *α* level of 0.05, and a statistical power (1 − *β*) of 0.90,and 19 predictor variables(Social support, acceptance of illness, fatigue,and clinical Variables). The sample size required to meet the demands of the multiple regression model was 187. Considering the potential inefficiency rate of 20%, the required number of participants was adjusted to 224. Accordingly, a total of 340 paper questionnaires were collected, and after screening, 317 valid questionnaires were obtained, yielding a response rate of 93.2%, which meets the modeling requirements.

### 2.2 Research tools

#### 2.2.1 General information questionnaire

After reviewing the relevant literature and discussing it with the research team, the researcher gathered the information (27–30). It includes gender, age, type of medical insurance, education level, marital status, pre-retirement occupation, living arrangement, number of comorbidities, smoking history, alcohol consumption history, fear of falling (self-reported: not afraid, slightly afraid, somewhat afraid, very afraid), Vision acuity status (self-reported: “Clear” vision within a 1-meter field of view; those with blurred or double vision were considered “blurred”), use of assistive walking devices (including crutches and walkers), and number of hospitalizations due to chronic diseases in the past year. Perceived Outdoor Environmental Comfort (self-reported: “comfortable,” “average,” or “uncomfortable”) (31). Self-care ability (assessed using a modified version of the Barthel Index Scale provided by the community healthcare system: ≤40 points indicates severe dependence, 41–60 points indicate moderate dependence, 61–99 points indicate mild dependence, and 100 points indicate no dependence) (32). In total, 16 variables were included.

#### 2.2.2 Social support rating scale (SSRS)

Developed by Xiao (33), it is widely used in China to assess social support. The scale contains 10 questions, organized into three categories: Subjective Support (4 items), Objective Support (3 items), and Support Utilization (3 items). The overall score ranges from 12 to 66, with higher scores reflecting stronger social support and better social connections. In this study, the Cronbach’s *α* coefficient for the SSRS was 0.825, indicating strong reliability and validity.

#### 2.2.3 Acceptance of illness scale (AIS)

Developed by Felton et al. (34), it is used to assess patients’ acceptance of illness-related feelings, such as uselessness, restriction, and dependence. It was translated into Chinese by Zhao (35). The scale consists of 8 items, is unidimensional, and uses a 5-point Likert scale, ranging from 1 (completely disagree) to 5 (completely agree). The total score ranges from 8 to 40, with higher scores reflecting a higher level of illness acceptance. A score of 30 above reflects a high level of disease acceptance, while scores between 20 and 29 suggest a moderate level, and scores ranging from 8 to 19 represent a low level. In this study, the Cronbach’s *α* coefficient for the AIS was 0.847.

#### 2.2.4 Multidimensional fatigue inventory (MFI)

Developed by Semets (36), it is a comprehensive tool designed to evaluate fatigue experienced by individuals over the past two weeks. It was adapted into Chinese by Miao et al. It was adapted into Chinese by Miao et al. (37), demonstrating good reliability and validity. The scale consists of 20 items across five dimensions: general fatigue (4 items), somatic fatigue (4 items), cerebral fatigue (4 items), reduced activity (4 items), and decreased motivation (4 items). Items 1, 3, 4, 6, 7, 8, 11, 12, 15, and 20 are scored inversely. A 5-level Likert scale is used, with scores ranging from 20 to 100, where higher scores indicate higher levels of fatigue. In this study, the Cronbach’s *α* coefficient for the scale was 0.863.

#### 2.2.5 Consumer health activation index (CHAI)

Developed by Wolf in 2015 (38), this assesses patients’ health activation levels concerning their disease. Cha translated it into Chinese and has demonstrated strong reliability and validity (39). The scale includes 10 items divided into three dimensions: Knowledge (3 items), Self-efficacy (4 items), and Action (3 items). It is rated on a 6-point Likert scale, with values ranging from 1 (strongly disagree) to 6 (strongly agree). The score ranges for each dimension are as follows: Knowledge (3-8 points), Self-efficacy (4-24 points), and Action (3-18 points). The initial total score ranges from 10 to 60. Using the score conversion table provided by the original authors, the initial total score was converted into a scale of 0 to 100, with higher values indicating greater levels of patient positivity. Based on the grading criteria, the scale is divided into three levels (39): scores between 0 and 79 reflect low self-management activation, scores from 80 to 94 indicate moderate activation, and scores between 95 and 100 denote high activation. In this study, the Cronbach’s *α* coefficient for this scale was 0.949.

### 2.3 Data collection and quality control

In compliance with the Helsinki Declaration, this study was approved by the Ethics Review Committee of the School of Nursing, Anhui Medical University (Approval No: 83241298). Before the study, permission and support were obtained from the person in charge of the community health center. Three nursing graduate students who had undergone standardized training used standardized instructions to explain the purpose, content, significance, and precautions for completing the questionnaire with older adult patients with chronic diseases. It was emphasized that participants had the right to withdraw from the study at any time and that withdrawal would not result in any adverse effects. After obtaining informed consent, the researchers distributed the questionnaires to the participants, who completed them independently and anonymously. For patients who had difficulty reading or writing and were unable to complete the questionnaire independently, the investigator provided objective explanations of the questionnaire items and accurately recorded their responses. We conducted a completeness check on all returned questionnaires to ensure data accuracy and clarify any missing data. The questionnaires were considered invalid if over 10% of the items were left unanswered, extreme values were repeatedly chosen, or multiple answers were selected for any multiple-choice question (40, 41). To protect participants’ privacy, all personal identification information (such as names and contact details) was removed, and all data was encrypted and securely stored. Only authorized researchers could access these data through a password-protected electronic system.

### 2.4 Statistical methods

This study used SPSS 26.0 for statistical analysis. Descriptive statistics were applied to present the demographic characteristics and core variables of the study subjects. Continuous variables were expressed as mean ± standard deviation (SD), and categorical variables were presented as frequency and percentage. Data normality was assessed using skewness and kurtosis, with data considered approximately normally distributed if their absolute values were within 3 standard deviations (42). Between-group comparisons were conducted using independent samples t-tests or one-way analysis of variance (ANOVA) for normally distributed data. The correlations between the study variables (social support, disease acceptance, fatigue symptoms, and self-management positivity) were analyzed using Pearson correlation analysis to explore the relationships between the variables. All tests were two-tailed, and the significance level was set at *p* < 0.05.

Additionally, AMOS 26.0 software was used to calculate 95% confidence intervals (CI) based on the Bootstrap method (5,000 samples) for conducting structural equation modeling (SEM) analysis. Through Bootstrap analysis, the significant effect of acceptance of illness and fatigue on the relationship between social support and self-management activation among older adult patients with chronic diseases was assessed. If the 95% CI does not include 0, it indicates that the indirect effect is statistically significant. After model optimization, the following fit indices were used to evaluate the model: chi-square/degrees of freedom (CMIN/DF, χ^2^/df < 3.00), root mean square error of approximation (RMSEA < 0.08), comparative fit index (CFI > 0.90), Goodness-of-Fit Index (GFI > 0.90), Normalized Fit Index (NFI > 0.90), Relative Fit Index (RFI > 0.90), Incremental Fit Index (IFI > 0.90), and Tucker-Lewis Index(TLI > 0.90). If the fit indices meet the criteria and *p* < 0.05, the model is considered well-fitted, and the best model is selected to ensure optimal explanatory power and stability (43).

## 3 Results

### 3.1 Common method deviation test

To minimize common method bias, implemented to mitigate its sources. Harman’s single-factor test was used in SPSS version 26.0 to conduct an exploratory factor analysis of all test items. The results showed that the first factor explained 38.501% of the variance, which is below the 40% threshold, indicating that there was no significant standard method bias in this study.

### 3.2 Demographic characteristics of participants

The total number of participants in this study was 317, with an average age of 74.56 ± 7.17 years. Among them, 51.4% of the participants were female, 52% had an educational level of Primary school or below, 57.7% lived only with their spouse, 41.3% reported not being afraid of falling, and 53.6% had a self-care ability score ranging from 61 to 99 (mild dependence). (See Table 1).

**Table 1:** Participants’ demographic characteristics (*n* = 317).

Variables	*n* (%)	Total score of self-management activation mean (SD)	*F*/*t*	*p*
Gender			1.936^△^	0.054
Male	154 (48.6)	71.23 (17.82)		
Female	163 (51.4)	67.44 (17.11)		
Age(years)			16.047	<0.001
65 ~ 69	91 (28.7)	75.98 (14.32)		
70 ~ 79	140(44.2)	69.69 (16.49)		
≥80	86 (27.1)	61.53 (19.30)		
Type of medical insurance			23.841	<0.001
Employee medical insurance	147 (46.4)	77.20 (14.35)		
Urban resident medical insurance	35 (11.0)	67.43 (18.49)		
New rural cooperative medical scheme	129 (40.7)	60.88 (16.86)		
Other medical insurance	6 (1.9)	69.67 (11.15)		
Educational level			32.754	<0.001
Primary school or below	165 (52.0)	62.48 (16.34)		
Junior high school	63 (19.9)	71.24 (18.39)		
High school	46 (14.5)	79.26 (12.67)		
Associate degree or above	43 (13.6)	81.81 (11.97)		
Marital status			31.875	<0.001
Married	258 (81.4)	72.71 (15.92)		
Divorced	6 (1.9)	49.67 (20.37)		
Widowed	53 (16.7)	54.83 (16.17)		
Pre-retirement occupation			33.436	<0.001
Worker	90 (28.4)	71.62 (16.42)		
Farmer	135 (42.6)	60.74 (16.73)		
Service personnel	84 (26.5)	79.38 (13.63)		
Other	8 (2.5)	81.00 (6.05)		
Living arrangement			16.412	<0.001
Living alone	35 (11.1)	56.23 (16.19)		
Living only with spouse	183 (57.7)	72.04 (16.26)		
Living only with children	32 (10.1)	57.69 (18.37)		
Living with spouse and children	67 (21.1)	74.09 (15.81)		
Number of comorbidities (Types)			13.169	<0.001
1	164 (51.7)	73.26 (15.91)		
2 ~ 3	141 (44.5)	66.11 (18.09)		
≥4	12 (3.8)	52.17 (15.36)		
Vision acuity status			5.848^△^	<0.001
Clear	213 (67.2)	73.34 (15.42)		
Blurry	104 (32.8)	60.96 (18.71)		
Use of assistive walking devices			−8.812^△^	<0.001
Yes	26 (8.2)	43.23 (18.55)		
No	291 (91.8)	71.61 (15.47)		
Number of hospitalizations due to chronic diseases in the past year(Number of times)			12.584	0.005
0	237(74.8)	71.61(16.19)		
1 ~ 2	77(24.3)	61.74(19.67)		
≥3	3(0.9)	78.67(4.16)		
Perceived outdoor environmental comfort			64.156	<0.001
Comfortable	144 (45.4)	78.92 (11.05)		
Neutral	159 (50.2)	62.18 (17.92)		
Uncomfortable	14 (4.4)	50.86 (14.77)		
Smoking history			−0.969^△^	0.333
Yes	45 (14.2)	66.93 (15.28)		
No	272 (85.8)	69.67 (17.88)		
Alcohol consumption history			0.160^△^	0.873
Yes	36 (11.4)	69.72 (18.27)		
No	281 (88.6)	69.22 (17.47)		
Fear of falling			31.704	<0.001
Not afraid	131 (41.3)	77.42 (12.28)		
Slightly afraid	80 (25.2)	70.60 (14.65)		
Somewhat afraid	64 (20.2)	63.16 (18.11)		
Very afraid	42 (13.3)	50.71 (18.66)		
Self-care ability (Points)			52.323	<0.001
41 ~ 60	10 (3.2)	39.40 (21.87)		
61 ~ 99	170 (53.6)	63.45 (16.10)		
100	137 (43.2)	78.70 (12.78)		

^△^Indicated as *t*-test.

Analysis of self-management activation about demographic characteristics revealed no significant differences (*p* > 0.05) for gender (*p* = 0.054), smoking history (*p* = 0.333), and alcohol consumption history (*p* = 0.873). However, significant differences (*p* < 0.05) were found for other demographic characteristics (Table 1).

### 3.3 Scores of social support, acceptance of illness, fatigue, and self-management activation in older adult patients with chronic diseases

Table 2 summarizes the descriptive statistics for each measured variable and the results of the multivariate normality test. Evaluation of the standard deviation, skewness, and kurtosis indicators confirmed that all variables followed a normal distribution. The self-management activation score was 69.28 ± 17.54, the average social support score was 39.77 ± 7.83, and the average fatigue score was 45.73 ± 10.85. Detailed statistical results for other dimensions are presented in Table 2.

**Table 2:** Scores of key variables in patients (*n* = 317).

Variable	Mean	SD	Skewness	Kurtosis
Social support	39.77	7.83	−0.52	−0.27
Subjective support	21.76	4.35	−0.70	−0.19
Objective support	10.29	2.27	−0.94	1.21
Utilization of support	7.72	2.49	0.04	−0.99
Acceptance of illness	28.04	6.46	−0.38	−0.67
Fatigue	45.73	10.85	0.62	0.08
General fatigue	9.71	3.10	0.44	−0.34
Physical fatigue	11.54	2.48	0.02	−0.51
Reduced activity	8.17	2.14	1.10	2.03
Reduced motivation	9.07	2.57	0.54	0.19
Mental fatigue	7.24	2.50	1.41	2.36
Self-management activation	69.28	17.54	−0.68	−0.29
Knowledge	19.94	6.33	−0.76	−0.27
Self-efficacy	29.13	6.49	−0.68	0.06
Action	20.21	6.00	−0.72	−0.15

### 3.4 Pearson correlation analysis

Table 3 presents the results of the Pearson correlation analysis, which showed that the self-management activation scores of older adult individuals with chronic conditions were significantly correlated with both social support and acceptance of illness (*r* = 0.615, 0.787, *p* < 0.01), as well as a significant negative correlation with fatigue (*r* = −0.695, *p* < 0.01).

**Table 3:** Correlation analysis of the relationship between self-management activation and key variables (*r* values).

Variable	Social support	Acceptance of illness	Fatigue	Self-management activation
Social support	1			
Acceptance of illness	0.641**	1		
Fatigue	−0.627**	−0.798**	1	
Self-management activation	0.615**	0.787**	−0.695**	1

**p < 0.01.

### 3.5 The mediating effect of acceptance of illness, fatigue on the relationship between social support and self-management activation among older adult patients with chronic diseases

**Figure 2:**
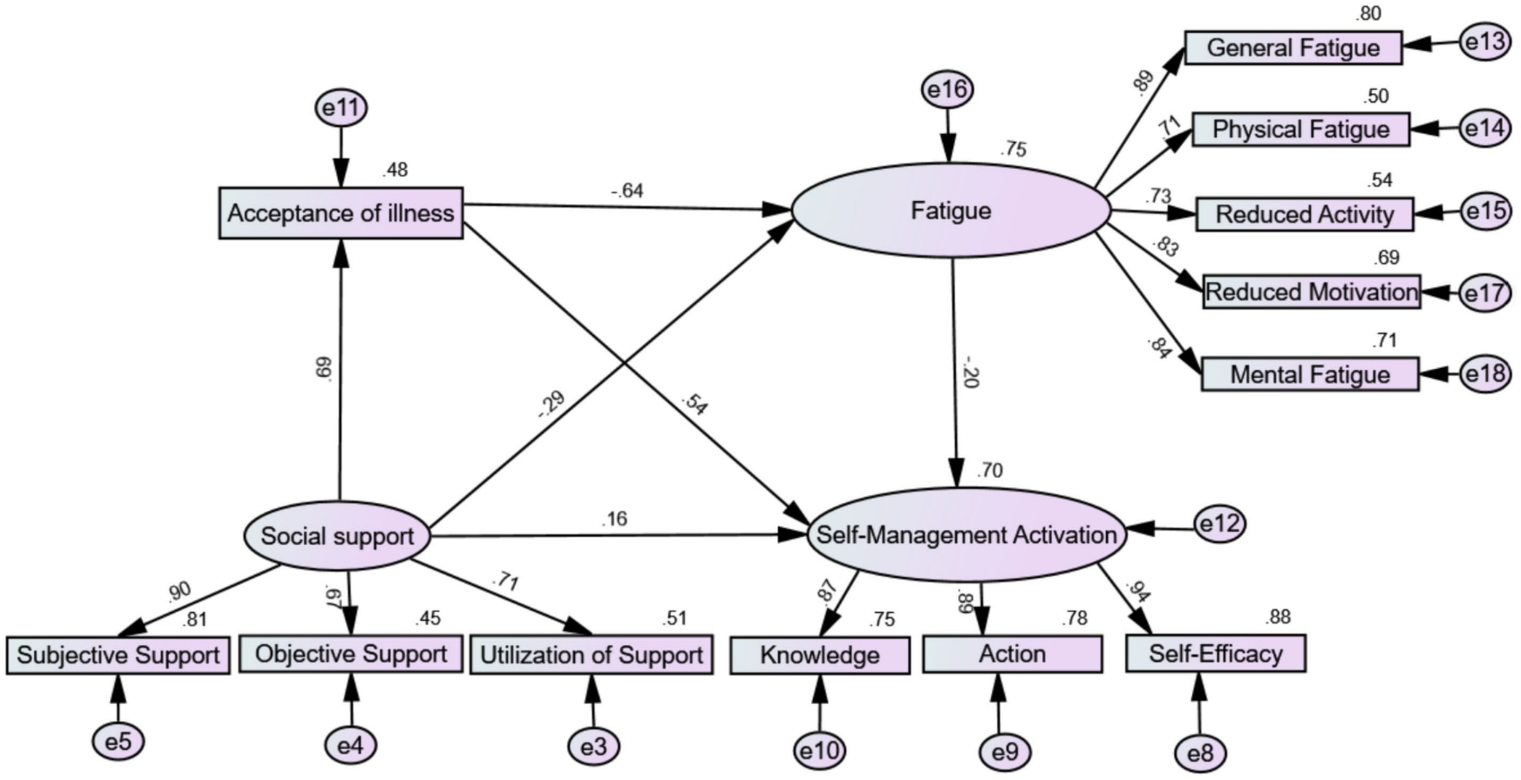
The chain mediating effect of social support and self-management activation.

To effectively control measurement errors, including acceptance of illness and fatigue symptoms, in the mediating effect between social support as the independent variable and self-management activation as the dependent variable, this study used a structural equation model to test the chain mediation effect. According to the mediation effect testing process proposed by Wen et al. (44), the SEM method in AMOS 26.0 software was used to validate the theoretical model. As shown in Figure 2, the model was refined using the maximum likelihood method and assessed for fit. Fit indices were then evaluated to determine the model’s adequacy: χ^2^/df = 2.814, RMSEA = 0.076, CFI = 0.970, GFI = 0.935, NFI = 0.954, RFI = 0.938, IFI = 0.970, and TLI = 0.959, indicating a good fit. Normalized path coefficients were computed to mitigate Type I errors arising from distributional issues. All standardized path coefficients were significant (*p* < 0.001). Social support had a significant positive predictive effect on acceptance of illness and self-management activation (*β* = 0.69, *p* < 0.001; *β* = 0.16, *p* < 0.001) and a significant negative predictive effect on fatigue (*β* = −0.29, *p* < 0.001). Acceptance of illness significantly positively predicted self-management activation (*β* = 0.54, *p* < 0.001), while fatigue significantly negatively predicted self-management activation (*β* = −0.20, *p* < 0.001).

To examine the chained mediating effects of acceptance of illness and fatigue on the relationship between social support and self-management activation, we employed a Bootstrap method with 5,000 repeated samples and calculated the 95% confidence intervals. The results are shown in Table 4, indicating that the mediating effects include three indirect pathways: Path 1: Social support → acceptance of illness → Self-management activation [effect = 0.371, 95% CI (0.265, 0.481)]; Path 2: Social support → fatigue → Self-management activation [effect = 0.056, 95% CI (0.016, 0.108)]; Path 3: Social support → acceptance of illness → fatigue → Self-management activation [effect = 0.087, 95% CI (0.021, 0.156)] The proportions of the indirect effects of these three paths account for 54.7%, 8.3%, and 12.8% of the total effect, respectively. The 95% confidence intervals for these indirect effects do not include 0, indicating that all mediating effects are significant.

**Table 4:** Results of mediation analysis between social support and self-management activation.

Effect	*β*	SE	95%CI		Relative mediation effect (%)
			Lower limit	Upper limit	
Direct effect	0.164	0.072	0.053	0.289	24.2%
Indirect effect	0.514	0.053	0.430	0.605	75.8%
Total effect	0.678	0.042	0.605	0.743	100%
1 → 2 → 4	0.371	0.066	0.265	0.481	54.7%
1 → 3 → 4	0.056	0.028	0.016	0.108	8.3%
1 → 2 → 3 → 4	0.087	0.041	0.021	0.156	12.8%

1, social support; 2, acceptance of illness, 3, fatigue; 4, self-management activation.

## 4 Discussion

With the rapidly aging population in China and the continuous rise in the incidence of chronic diseases among the older adult, the activation of self-management has become a key factor in improving the quality of life for patients. This study examined the association between social support and self-management activation among older adult patients with chronic diseases, with particular emphasis on the mediating roles of illness acceptance and fatigue in this process. The results demonstrated that self-management activation was significantly and positively correlated with social support and illness acceptance, while it was negatively correlated with fatigue. Furthermore, social support not only directly and positively correlated with self-management activation but also further enhanced it through the independent and combined mediating effects of illness acceptance and fatigue. These findings suggest that improving illness acceptance and alleviating fatigue are crucial for strengthening self-management activation among older adult patients with chronic diseases, providing new insights and directions for future initiatives aimed at improving self-management activation.

### 4.1 The relationship and current status of social support and self-management activations

The study results indicate that the social support scores of older adult patients with chronic diseases were 39.77 ± 7.83, reflecting a moderately high level, which was higher than the results reported in previous studies (45, 46). This may be attributed to the fact that 67.2% of the respondents in this study had clear vision, 43.2% did not require assistance with activities of daily living, and 91.8% did not need mobility aids, suggesting that most patients maintained a high level of independence in daily living. As a result, they were more likely to actively seek social support to enhance their confidence in managing their illnesses (47). Additionally, 41.3% of patients did not fear falling, and 45.4% held a positive perception of outdoor environments, indicating greater confidence in their physical abilities and a stronger inclination to engage in outdoor activities and social interactions, which may be related to higher levels of social support (48).

The study also revealed that the self-management activation scores of community-dwelling older adult patients with chronic diseases were 69.28 ± 17.54, indicating a low level. This finding is consistent with the results of Zhu et al. (24) on older adult patients with coronary heart disease. This suggests that older adult patients with chronic diseases generally demonstrate insufficient willingness to engage in health-promoting behaviors during disease management. Several factors may contribute to this: first, misconceptions about the disease may lead patients to overly rely on medical technology while underestimating the critical role of lifestyle and behavioral management, thus weakening their proactive self-management. Second, 48.3% of the respondents had two or more chronic diseases. Comorbidity not only increases the risk of frailty and adverse health outcomes (49), but also may exacerbate psychological burdens, leading patients to feel helpless and prefer passive treatment over active disease management. Furthermore, the complex requirements of disease management, such as long-term polypharmacy, strict dietary control, and frequent health monitoring, increase the implementation burden on patients, thereby triggering both physiological and psychological stress (50). Over time, this burden may lead to anxiety, fatigue, and even resistance toward health management, further diminishing self-management activation.

High levels of social support can directly enhance patients’ confidence in managing their disease, which is consistent with previous studies (51). Social support, by providing necessary information, economic assistance, and emotional support, not only directly influences patients’ motivation for disease management but also serves a buffering role in alleviating emotional disorders, effectively mitigating the impact of adverse stress events, and injecting external motivation into patients (52). Additionally, this study further found that the mediating roles of illness acceptance and fatigue account for 75.8% of the indirect effect between social support and self-management activation. This finding underscores that patients’ self-management activation is not only dependent on external social support but also requires substantial internal factors (53). Therefore, to further enhance the promotional role of social support in self-management activation, it is essential to focus on patients’ psychological adaptation and physiological status, optimize the allocation of community health resources, and improve patients’ awareness and utilization of social resources. Such efforts would effectively convert external support into actual health management behaviors, ultimately improving health outcomes.

### 4.2 Mediating role of acceptance of illness

Research findings indicate that acceptance of illness functions as a critical mediating variable between perceived social support and self-management engagement among older adult patients with chronic illnesses. Consequently, Hypothesis 2 is supported. This aligns with prior empirical evidence suggesting that higher levels of social support enhance patients’ sense of security and belonging, facilitating the reappraisal of health threats, promoting acceptance of illness, reducing psychological resistance, and improving health management awareness (12). Moreover, elevated acceptance of illness not only strengthens health identity but also stimulates intrinsic motivation for self-management, encouraging patients to view their chronic condition as manageable and to adopt proactive health strategies. In contrast, patients with low acceptance of illness are more prone to negative emotional states such as anxiety, depression, and self-negation (20), which diminish the positive effects of social support and reduce health management intentions and adherence. Acceptance of illness emerges as a pivotal psychosocial regulatory factor, significantly influencing disease management behaviors and psychological well-being (9). Patients with high acceptance of illness typically employ adaptive coping mechanisms, effectively adjusting to their illness and social environment, thereby demonstrating greater self-management activation. For patients with low acceptance of illness, interventions that integrate psychological counseling, patient education, and peer support are recommended to foster positive illness perceptions and strengthen adherence to health behaviors. Additionally, community healthcare providers should emphasize the long-term impact of acceptance of illness on health management during health education and follow-up, thereby enhancing patient confidence in disease control.

### 4.3 The mediating role of fatigue

The study results indicate that fatigue plays a partial mediating role in the relationship between social support and the self-management activation of older adult patients with chronic diseases. Therefore, Hypothesis H3 is validated. Specifically, patients without fatigue, due to their better mental state, can utilize assistance from external resources more effectively, such as society and family, converting this support into motivation for health management. This transformation enhances patients’ abilities to cope with their disease, thereby increasing their willingness and energy to engage in health management activities such as health monitoring, medication management, and lifestyle adjustments. Conversely, research indicates that patients with higher levels of fatigue often struggle to maintain positive health management behaviors, as physical fatigue leads to reduced motivation, emotional exhaustion, or diminished self-efficacy (54). Mental fatigue is a significant physiological and psychological barrier that negatively impacts the ability to activate self-management. It not only significantly weakens patients’ health behaviors and quality of life but also exacerbates physical decline and reduced activity caused by the overlap of disease symptoms and physical function deterioration, making daily health management tasks more difficult (55). Additionally, cognitive fatigue impairs patients’ cognitive abilities and emotional regulation, thereby complicating health decision-making and the implementation of behavioral changes. Therefore, for patients experiencing severe fatigue, multidimensional comprehensive intervention measures, such as exercise interventions and psychological regulation, should be implemented to alleviate both physical and mental fatigue and enhance health management capabilities. Furthermore, healthcare professionals should prioritize fatigue management during community health education and follow-up visits, implementing personalized intervention plans to maximize the positive effects of social support, thereby improving self-management activation among older adult patients with chronic diseases and enhancing their overall health outcomes.

### 4.4 The chain mediating effect of acceptance of illness and fatigue

The results of this study indicate that social support exerts its effects through a chain of mediating pathways involving acceptance of illness and fatigue. Therefore, Hypothesis H4 is supported. This finding aligns with the cognitive-somatic integration model (56), which suggests that positive cognitive perceptions of the disease may reduce emotional exhaustion, thereby lowering fatigue levels and ultimately enhancing self-management activation. It highlights the relationship between psychological adaptation and physiological status, offering new insights for intervention strategies.

In summary, as China’s family structure becomes increasingly nuclearized, the proportion of older adult individuals in empty nests continues to rise, leading to a decline in emotional support and companionship within families. Combined with the long-term burden of chronic diseases and physical decline, the older adult’s ability to engage in outdoor activities is limited, further diminishing their level of social support. The prolonged lack of social support makes patients more vulnerable to feelings of isolation and helplessness during disease management, depriving them of sufficient emotional comfort and practical assistance. This not only affects their health perceptions but also weakens their acceptance of illness. Acceptance of illness is closely related to fatigue (21). Patients with low disease acceptance tend to avoid confronting the reality of their illness and lack intrinsic motivation for health management, thereby exacerbating both psychological and physical fatigue. The presence of fatigue not only reduces patients’ willingness to engage in health management activities but also negatively impacts their health cognition and decision-making abilities, significantly hindering the implementation of health behaviors and ultimately leading to a decline in self-management motivation. Therefore, acceptance of illness not only directly influences self-management activation but also alleviates the adverse effects of fatigue by improving emotional states and enhancing health cognition, thereby promoting the implementation of health behaviors (57). Simply increasing acceptance of illness may still be insufficient to enhance self-management levels significantly; without concurrent fatigue management, the execution of health behaviors may remain limited.

Therefore, when developing health management programs, it is essential to systematically consider the synergistic effects of social support, acceptance of illness, and fatigue. Particular attention should be given to maximizing the health-promoting effects of social support by enhancing acceptance of illness while integrating fatigue management strategies to optimize patients’ health management capabilities. Community healthcare providers should adopt comprehensive measures in practical interventions, such as personalized health education, peer support, cognitive-behavioral interventions, and physical rehabilitation training, to improve patients’ acceptance of their illness. Additionally, effective fatigue management strategies should be implemented, such as exercise rehabilitation, psychological adjustment, and sleep optimization. By reducing the inhibitory effects of fatigue on health behaviors while enhancing patients’ self-efficacy and health management beliefs, these approaches ultimately help patients improve their self-management capabilities and achieve better health outcomes.

## 5 Limitations

This study has several limitations. First, Although the target population was older adults with chronic diseases in China, the sample was drawn through convenience sampling from three urban communities in Hefei. As such, it may not fully reflect older adult populations in other regions, particularly rural areas, due to differences in socioeconomic status, healthcare access, and cultural attitudes toward self-management. This limits the external validity of the findings. Given China’s large population and regional diversity, future studies should recruit more representative samples from various provinces and both urban and rural settings to improve generalizability. Second, although this study effectively explored the associations between social support, acceptance of illness, fatigue, and self-management activation, the cross-sectional design used in this study prevents the identification of causal relationships among the variables. To better understand the dynamic changes and causal pathways among these variables, future studies should consider using a longitudinal design to track how variables change over time, thereby further validating the causal relationships among them. Finally, although this study employed self-report measurement tools to simplify the data collection process, this approach may be influenced by social desirability effects or privacy concerns, leading participants to inaccurately report their situations, which could result in reporting bias. To reduce this bias, future studies could employ a combination of multiple data collection methods, such as physiological indicator testing, peer assessment, or behavioral observation, to provide more objective data support and enhance the reliability and validity of the results.

## 6 Conclusion

Previous studies have primarily focused on examining variables associated with self-management activation through multiple logistic regression analysis, with a limited in-depth investigation into the intrinsic relationships among variables. Mediating effect analysis can reveal the direct and indirect influences between variables and explain their intrinsic connections. This study is the first to identify the acceptance of illness as a key mediating factor whose enhancement can significantly mitigate the negative impact of fatigue and promote self-management activation. Acceptance of illness and fatigue plays a mediating role in the chain between social support and self-management activation, thereby validating our research hypothesis and further illustrating the synergistic role of psychological and physiological factors in health management. These findings not only further validate and enrich the application of social cognitive theory in the field of older adult chronic disease management but also provide a scientific basis and empirical support for improving intervention strategies to enhance self-management activation among older adult chronic disease patients. Future research should focus on enhancing acceptance of illness, alleviating fatigue, and strengthening the integrated role of social support in managing chronic conditions. By combining multidisciplinary interventions (such as rehabilitation, nutrition, mental health, and family support), diverse comorbidity management strategies should be explored to comprehensively improve patients’ self-management capabilities and enhance their health outcomes.

## Data Availability

The original contributions presented in the study are included in the article/supplementary material, further inquiries can be directed to the corresponding author.
